# Basidiomycota species in Drosophila gut are associated with host fat metabolism

**DOI:** 10.1038/s41598-023-41027-2

**Published:** 2023-08-23

**Authors:** Berkay Bozkurt, Gamze Terlemez, Efe Sezgin

**Affiliations:** 1 Bioengineering Program, Izmir Institute of Technology, Urla, Izmir Turkey; 2 Biotechnology Program, Izmir Institute of Technology, Urla, Izmir Turkey; 3 Department of Food Engineering, Izmir Institute of Technology, Urla, Izmir Turkey

**Keywords:** Microbial communities, Fungi, Genetic association study, Genomics, Quantitative trait

## Abstract

The importance of bacterial microbiota on host metabolism and obesity risk is well documented. However, the role of fungal microbiota on host storage metabolite pools is largely unexplored. We aimed to investigate the role of microbiota on *D*. *melanogaster* fat metabolism, and examine interrelatedness between fungal and bacterial microbiota, and major metabolic pools. Fungal and bacterial microbiota profiles, fat, glycogen, and trehalose metabolic pools are measured in a context of genetic variation represented by whole genome sequenced inbred Drosophila Genetic Reference Panel (DGRP) samples. Increasing Basidiomycota, *Acetobacter persici*, *Acetobacter pomorum*, and *Lactobacillus brevis* levels correlated with decreasing triglyceride levels. Host genes and biological pathways, identified via genome-wide scans, associated with Basidiomycota and triglyceride levels were different suggesting the effect of Basidiomycota on fat metabolism is independent of host biological pathways that control fungal microbiota or host fat metabolism. Although triglyceride, glycogen and trehalose levels were highly correlated, microorganisms’ effect on triglyceride pool were independent of glycogen and trehalose levels. Multivariate analyses suggested positive interactions between Basidiomycota, *A. persici*, and *L*. *brevis* that collectively correlated negatively with fat and glycogen pools. In conclusion, fungal microbiota can be a major player in host fat metabolism. Interactions between fungal and bacterial microbiota may exert substantial control over host storage metabolite pools and influence obesity risk.

## Introduction

Vertebrates and invertebrates coexist with a rich microbiota consisting of bacteria, fungi, archaea, and viruses. The gastrointestinal tract is one of the richest microbiota ecosystems on the organism^1^. Gut microbiome can influence the host metabolism and nutrition status^2–4^, gut development and gut-brain connection^5,6^, and immunity^7,8^. Microbiota can also modulate endothelial tissue repair, regulation of intestinal endocrine functions^9^, energy production, weight gain and development of insulin resistance^10^.

Obesity is associated with altered bacterial microbiota composition where bacterial diversity reduction in human and animal models are reported^11–16^. Whether microbiota composition change drives obesity or it is just a result of host metabolic change is still not clear^17^. However, reports suggest obesity related microbiota can extract calories from indigestible polysaccharides more efficiently^11,18,19^, can alter host metabolism^14,20^, and influence satiety related behavior^21^.

Nearly all microbiota studies focus on prokaryotic organisms (such as bacteria). Despite their much larger genome, thus possibility of more complicated interactions with the host, the effect of fungal microbiome (also called the mycobiome) on host metabolism is largely unexplored. Most fungal-host interactions focus on infectious diseases, however, changes in fungal microbiome has also been reported in obesity^22^, autoimmune conditions^23^, neurologic conditions^24^, cancer^25^, and mycobiome may influence bacterial microbiota composition^26^. Therefore, further research on mycobiome-host interactions is needed.

Over the last decade *Drosophila melanogaster* has emerged as a model organism to mechanistically study host-microbiota intercations. Although *D. melanogaster* has a much simpler bacterial microbiota compared to vertabrates^27,28^, its microbiome has profound physilogical effects on the host such as larval development, nutrition status, metabolism, immune regulation, and aging^4,29–33^. Moreover, as *D. melanogaster* is an excellent model for controlled experiments and amenable to genetic manipulation. With the D. melanogaster model, studying correlations between microbiota and host metabolism may promote the identification of the underlying biological pathways more easily ^32,34–36^. Similar to other organisms the fungal microbiome studies in *Drosophila* is rather limited. The fungal microbiome of *Drosophila* differs based on host species, geography and nutrition status^37–42^. Some fungal species are utilized as energy and protein source by *Drosophila*, and reported to influence host fecundity, fertility, and life span^43^. These observations suggest that host-fungal microbiome interactions may influence host metabolism and physiology in *Drosophila*.

In this study we primarily aimed to investigate the role of fungal microbiota on *D*. *melanogaster* fat and glycogen metabolism, the main energy storage metabolites. Trehaolose is the circulating blood sugar in insects. We also aimed to examine the correlations between bacterial microbiota and these metabolic pools, and quantify interrelations between fungal and bacterial microbiota, and fat and glycogen pools. To address these aims, we first identified the fungal and bacterial microbiota in a subset of *D*. *melanogaster* Drosophila Genetic Reference Panel (DGRP)^44^ samples. DGRP lines are whole genome sequenced inbred lines derived from natural populations. They are a widely used community resource for studying genetic architecture of complex phenotypes^44^. Then, we quantified the relative amount of most common fungal and bacterial taxa in 120 DGRP lines. Fat, glycogen, and trehalose metabolic pools are also measured in these DGRP samples. The relationships between the fungal and bacterial taxa, and the metabolites are examined.

## Materials and methods

### *Drosophila melanogaster* (DM) stocks

120 Drosophila Genetic Reference Panel (DGRP) stocks^44,45^, purchased from Bloomington Drosophila Stock Center (Bloomington, Indiana, USA), were used. The stocks were grown on a standard medium^46^ consisting of optimized agar-corn-sugar-non-living yeast extract. Similar egg and larval density was maintained in each vial. Stocks were kept in a climate-controlled room at 25 °C and relative humidity of 65% on a 12-h day and night cycle.

### Bacterial and fungal DNA isolation from fly gut samples

Five to seven days old five adult male flies from each DGRP line were collected. Each fly sample was washed with 10% sodium hypochlorite followed by distilled sterile water for surface sterilization repeating the washing process three times. The intestinal tube was removed by separating the abdomen from thorax with a sterile scalpel and tweezers in sterile Ringer's solution. DNA isolation was performed by the High Pure PCR Template Preparation kit (Roche Applied Science, Germany). Five intestinal samples belonging to each DGRP lines were placed in eppendorf tubes, 200 μl of tissue lysis buffer and 40 μl of proteinase K were added, mixed well crashing the tissues. After incubation at 55 °C for 1 h, 10 µl of lyticase (for fungal DNA) or 5 µl of lysozyme (for bacterial DNA) is added and incubated at 37 °C. Incubation period was 30 min for fungi and 15 min for bacteria. DNA binding, washing and elution steps were continued according to the manufacturer’s protocol. The concentration (ng/μl) and purity (A260/A280 and A260/A230 ratios) of the DNA samples were determined by NanoDrop 8000-C Spectrophotometer (Thermo Fisher Scientific, USA). The presence of fungal and bacterial DNA was tested with randomly selected samples. The PCR reactions were performed by ITS (ITS1-F: TCCGTAGGTGAACCTGCGG and ITS4-R: TCCTCCGCTTATTGATATGC) and 16S rRNA gene (331-F: TCCTACGGGAGGCAGCAGT and 797-R: GGACTACCAGGGTATCTAATCCTGTT) primers utilizing the FastStart High Fidelity PCR System, dNTPack kit (Roche Applied Science, Germany) according to the manufacturer protocol. All samples were kept at − 80 °C until further processing.

### Identification of fungal and bacterial microbiota: ITS region and 16S rRNA gene amplicon sequencing

For fungal and bacterial microbiota determination ITS region and 16S rRNA genes were targeted and amplified by specific primers listed in Supplemental Table 1 for ten randomly selected DGRP lines (line numbers 138, 217, 235, 26, 354, 370, 439, 705, 837, 900 represented by S1–S10 in Supplemental Figs. 1 and 2). After quantification and qualification of PCR products, sequencing libraries were generated with Nextera XT DNA Library Preparation Kit (Illumina, USA). Concentration of the libraries were normalized by diluting to 4 nM then paired-end (2 × 250 bp) sequenced by Illumina NovaSeq 6000. Raw data quality and quality control of the reads were checked by FastQC and QIIME2, respectively. Primer and barcode sequences, chimeric reads, and reads with Phred Score less than 20 were removed by DADA2 and effective tags were obtained. QIIME2 was used for taxonomic determination of each Operational Taxonomic Units (OTUs). Representative sequences for OTUs with ≥ 97% similarity against the Greengenes (version 13_8)^47^ and SILVA (SILVA 138.1)^48^ for bacteria, and UNITE (version 8.3)^49^ databases for fungi were generated. Rarefaction curves plotting sequencing depth vs. number of taxa identified were used to judge the appropriateness of sequencing depth for each sample^50,51^. Most abundant OTU representative sequences were also blasted and compared with the reference sequences in National Center for Biotechnology Information (NCBI).

### Quantification of selected fungal and bacterial taxa levels using real-time quantitative PCR (qPCR) analysis

Following the fungal and bacterial microbiota analyses in 10 randomly selected DGRP samples, the presence of the common bacterial species and fungal phyla in 120 DGRP strains and their relative abundance were determined by qPCR. DNA extraction from five guts of each DGRP line was performed as described above. Primers used to assay selected species/taxa are listed in Supplemental Table 2. Primers were designed by IDT SciTools OligoAnalyzer 3.1 (https://www.idtdna.com/calc/analyzer) software and specificity of primers were checked with BLAST (https://www.ncbi.nlm.nih.gov/tools/primerblast/). For a single reaction, 0.5 ng template DNA, 1.9 µl of PCR-grade water, 0.3 µl (10 pmol/µl concentration) of microorganism specific forward and reverse primers, and 5 µl of LightCycler^®^ 480 SYBR Green I Master enzyme (Roche Applied Science, Germany) were mixed and total volume was adjusted to 10 µl. Distilled water was used as a negative control in each run. Positive controls included *Saccharomyces cerevisiae* for Ascomycota, *Agaricus bisporus* for Basidiomycota, and cultured pure strains of *Acetobacter persici*, *Acetobacter pomorum*, *Enterococcus faecium*, *Lactiplantibacillus brevis* (formerly known as *Lactobacillus brevis*), and *Lactiplantibacillus plantarum* (formerly known as *Lactobacillus plantarum*). All reactions were carried out in duplicates on a Roche LightCycler^®^ 480 II Real-Time PCR System to the following conditions: 95 °C for 10 min with initial denaturation followed by 50 cycles of 95 °C for 10 s, annealing at temperatures showed in Supplemental Table 2 for 15 s, and 72 °C for 15 s. Melting curve analysis was used to assess the success of qPCR reactions with a ramp rate of 0.11 °C/s. Amplification and melting curves for each sample were obtained from Absolute Quantitation/Second Derivative and Tm Calling analysis modes in the LightCycler^®^ 480 II Software v.1.5. Relative abundance of target species/phyla was expressed as 2^-ΔCt^ and calculated according to the following formulas^52,53^.

Relative abundance of target fungal phylum with respect to abundance of total fungi:

$$ {2}^{{ - \Delta {\text{Ct}}}} \, = \,{2}^{{ - ({\text{Ct of target fungal taxa}}{ - }{\text{Ct of total fungi}})}} . $$

Relative abundance of target bacterial species with respect to abundance of total bacteria:

$$ {2}^{{ - \Delta {\text{Ct}}}} \, = \,{2}^{{{-}({\text{Ct of target bacteria}}{ - }{\text{Ct of total bacteria}})}} . $$

### Metabolic pool assays

Two flies from each DGRP strain were homogenized in 300 µl of NP40 Substitute Assay Reagent (Cayman, Item No. 700024) buffer solution and then centrifuged for 10 min at 10,000*g* in 4 °C. After separating 20 µl of homogenate for the protein assay, the remaining supernatant was denatured at 70 °C for 15 min for glucose, glycogen, trehalose and triglyceride measurements. Each metabolic pool was measured in duplicates, and the mean of results were used in further analyses.

Glucose, glycogen, and trehalose levels were measured using a commercially available kit (Cayman Glucose Colorimetric Assay Kit, Item No. 10009582). For glycogen amount calculation the glycogen is digested into free glucose. Fifteen μl of fly homogenate samples including 5 μl of 250 mM Na phosphate assay buffer (pH 7.2), were enzymatically digested by amyloglucosidase (A1602 from Sigma, St. Louis) at 37° for 1 h. Then glucose levels were measured using 15 μl of digested homogenate in 200 μl of assay solution by Cayman Glucose Colorimetric Assay Kit (Item No. 10009582). Glycogen (Cayman 700481) standard is used for preparation of standards at different concentrations, and they were subjected to enzymatic digestion process together with the samples. Glucose standard curve was used to determine glucose levels within samples. Then, by subtraction the absorbance of glucose in the untreated samples from the absorbance of samples diluted with amyloglucosidase, glycogen levels were calculated based on the glycogen standard curve. For trehalose amount calculation, 15 μl of fly homogenate samples containing 5 μl of 250 mM Na phosphate assay buffer (pH 7.2), were enzymatically digested by trehalase (T8778 from Sigma, St. Louis) at 37° for 24 h. Then glucose levels were measured using 15 μl of digested homogenate in 200 μl of assay solution by Cayman Glucose Colorimetric Assay Kit (Item No. 10009582). Trehalose (Cayman 20517) standard was taken for trehalose analysis, standards were prepared at different concentrations, and they were subjected to enzymatic digestion process together with the samples. Glucose standard curve was used to determine glucose levels within samples. Then, by subtraction the absorbance of glucose in the untreated samples from the absorbance of samples diluted with trehalase, trehalose levels were calculated based on the trehalose standard curve. Triglyceride (TGA) was measured with a commercially available kit (Cayman Triglyceride Colorimetric Assay Kit, Item No. 10010303). Ten microliters of fly homogenate was mixed with 150 μl of assay solution and incubated at 37° for 30 min. Protein concentration was measured based on the Bradford method as implemented in the Protein Determination Kit (Cayman, ltem No. 704002) according to the manufacturer protocol. Ten μl of fly homogenate was diluted to 500 μl with ddH_2_0. 100 μl of the diluted fly homogenate was incubated with 100 μl of protein determination kit reagent at room temperature for 5 min. Standard curves and linear regression formulas, constructed by plotting absorbance versus increasing prepared concentrations of each metabolite, were used for quantification of metabolites in each sample. All metabolite measurements were standardized by protein amount and reported as milligrams of metabolite per milligrams of protein.

### Genome-wide association studies (GWAS) and annotation of candidate genes

Genes and genetic variants associated with triglyceride, basidiomycote, *A. persici*, *A. pomorum*, and *L. brevis* levels were identified with the DGRP reference panel GWAS web tool (http://dgrp2.gnets.ncsu.edu/index.cgi). Mixed effect linear models included wolbachia status and chromosomal inversions in individual fly genomes. Only genes and variants with P < 10^–5^ are considered biologically relevant, and used for further annotation. Flybase (https://flybase.org/) and NCBI (https://www.ncbi.nlm.nih.gov/) were used for functional annotation of genes identified in the GWAS. STRING (https://string-db.org/) web tool was used to examine protein network interactions, GO annotations, biological pathway and functional enrichment tests.

### Statistical analysis

Shapiro–Wilk's test was used to test the normality assumption of the data. The bestNormalize package in R (https://cran.r-project.org/web/packages/bestNormalize/index.html) was used to normalize the data. Pearson correlation coefficient was utilized to investigate the relationships between dominant fungal and bacterial microbiota taxa, and metabolite variables. Univariate and multivariable regression analyses were used to statistically model the effect of examined taxa on metabolic pools. Pairwise significant correlations between several microbiota and metabolite variables was evident. Canonical correspondence analysis (CCA) was used to formulate the significant linear combinations that elucidate the most influential correlations between microbiota and metabolite variables. CCA (https://cran.r-project.org/web/packages/CCA/CCA.pdf) and CCP (https://cran.r-project.org/web/packages/CCP/CCP.pdf) R packages were used to conduct canonical correspondence analyses. All statistical analyzes were performed with R software (Version 4.2.2) (https://www.r-project.org). P-values less than 0.05 was accepted as statistically significant.

## Results

### Identification of fungal microbiota profile in the *Drosophila* gut

An average of 122,684 reads per sample (range 60,766–253,297) were generated for the nine DGRP gut samples. In all sequences, three phyla, 15 classes, 46 orders, 87 families, and 113 genus were identified based on a 97% similarity criteria. The most abundant phyla in the samples were Ascomycota (97.5%), Basidiomycota (2.4%), and Mucoromycota (0.1%) (Supplemental Fig. 1). Saccharomycetes was the dominant fungi class followed by Dothideomycetes within *Drosophila* gut, cumulatively representing over 95% of the ITS sequences in our samples. As species level identification was not possible for most reads, we targeted all Ascomycota and Basidiomycota taxa using the primers listed in Supplemental Table 2 and quantified the relative abundance of Ascomycota and Basidiomycota in 120 DGRP samples by qPCR to be used in further analyses.

### Identification of bacterial microbiota profile in the *Drosophila* gut

An average of 129,585 reads per sample (range 47,575–236,311) were generated for the ten DGRP gut samples. In all sequences, six phyla, 19 classes, 42 orders, 93 families, 276 genus, and 614 species were identified based on a 97% similarity criteria. The most abundant phyla in the samples were *Firmicutes* (70.5%), *Proteobacteria* (21.8%), *Bacteroidetes* (4.8%), *Actinobacteria* (2.7%), and *Fusobacteria* (0.1%) (Supplemental Fig. 2). For most reads accounting over 5% of the total number of reads species level identification was possible. We targeted relatively common identified species (*Acetobacter persici*, *Acetobacter pomorum*, *Enterococcus faecium*, *Lactiplantibacillus brevis*, and *Lactiplantibacillus plantarum*) that were also reported in previous Drosophila studies using the primers listed in Supplemental Table 2, and quantified the relative abundance of these species in 120 DGRP samples by qPCR to be used in further analyses.

### Metabolic pool profiles in the DGRP lines

Glucose, glycogen, trehalose, and triglyceride levels were quantified as milligram metabolite per milligram of protein. The mean (± SD) and median (25%,75%) glucose measurements were 0.19 (± 0.12) and 0.18 (0.12, 0.23). For glycogen, trehalose, and triglycerides the mean (± SD) and median (25%,75%) measurements were 0.36 (± 0.44) and 0.20 (0.09, 0.47), 0.03 (± 0.03) and 0.02 (0.01, 0.04), 1.03 (± 0.65) and 0.84 (0.59, 1.33), respectively. The distributions of all metabolites significantly deviated from normality (Supplemental Fig. 3, Shapiro–Wilk’s test p-values < 0.001). Therefore, normalized metabolic measurements were used in correlation and association analyses.

### Relationships between fungal and bacterial microbiota, and metabolic pools

There was a positive significant correlation between trehalose, glycogen, and triglyceride metabolite levels, where samples with higher triglycerade levels also had higher glycogen and trehalose levels, or vice versa (r: 0.41–0.48, Ps < 0.01; Supplemental Fig. 4). Moreover, a significant negative correlation between Basidiomycota and triglyceride levels was observed (r: −0.22, P < 0.05; Supplemental Fig. 4). Basidiomycota level did not show a strong correlation with other metabolites. Also, Ascomycota level did not show strong correlation with any of the metabolites (Supplemental Fig. 4).

*A*. *persici* level showed positive correlation with all other bacterial species examined (r: 0.24–0.74, Ps < 0.05; Supplemental Fig. 5), whereas *L*. *brevis* showed positive correlation with *A*. *pomorum* (r: 0.47, P < 0.001), and *E*. *faecium* (r: 0.22, P < 0.05; Supplemental Fig. 5). Negative correlations between *A*. *pomorum* and triglyceride (r: −0.72, P < 0.001), glycogen (r: −0.35, P < 0.001), and trehalose (r: −0.33, P < 0.001) levels was observed (Supplemental Fig. 5). Triglyceride level was also negatively correlated with *A*. *persici* (r: −0.27, P < 0.05) and *L*. *brevis* (r: −0.29, P < 0.01) levels. In addition, negative correlation between *E*. *faecium* and glycogen levels (r: −0.33, P < 0.001) was observed (Supplemental Fig. 5).

Examination of associations between fungal and bacterial taxa showed positive correlations between Basidiomycota and *A*. *persici* (r: 0.28, P < 0.01), and *A*. *pomorum* (r: 0.21, P < 0.05) levels (Supplemental Fig. 6). A weak positive correlation between total fungi and total bacteria levels was also observed (Supplemental Fig. 6).

**Figure 1:**
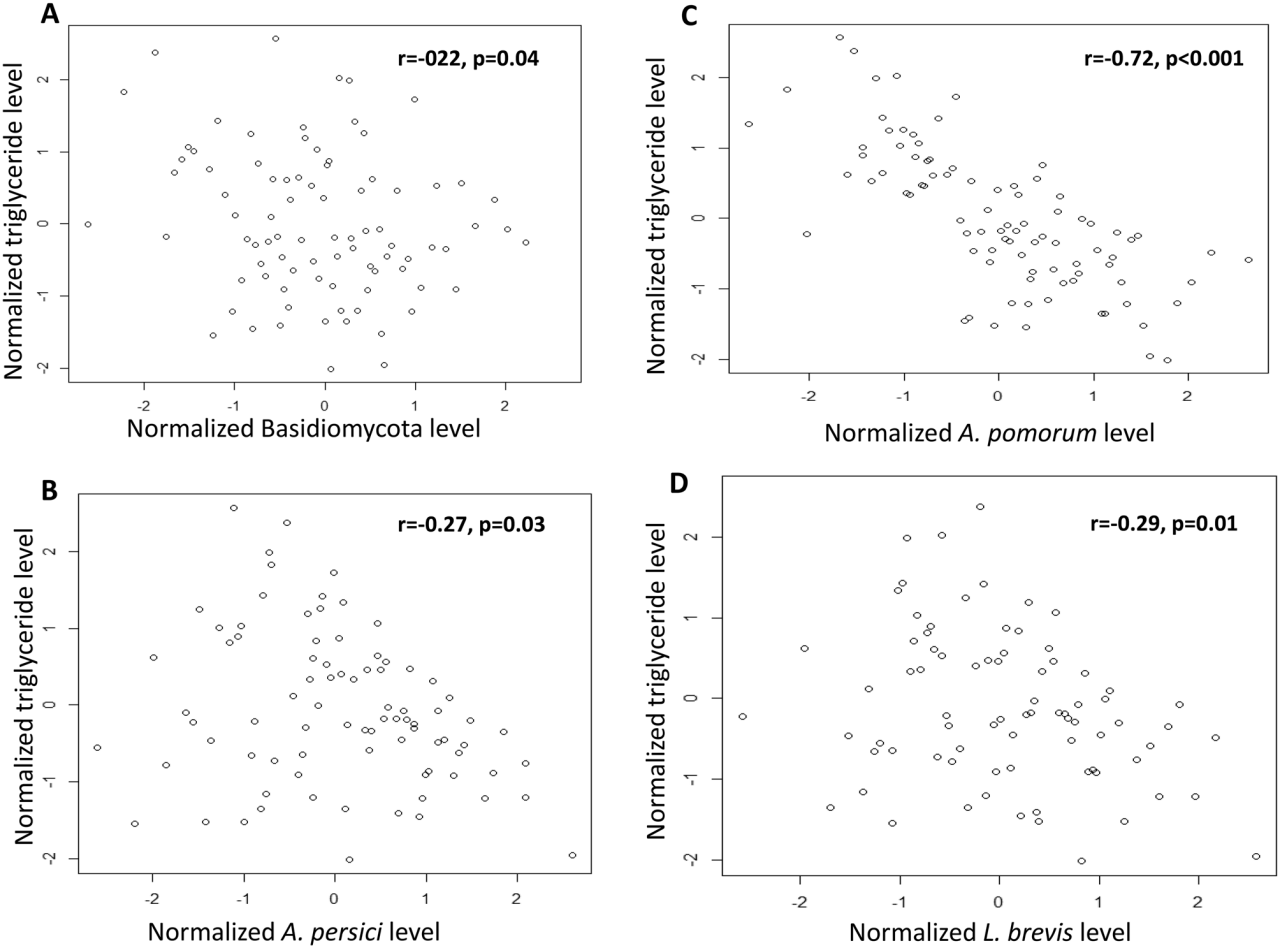
Scatterplots of normalized triglyceride level versus (**A**) normalized Basidiomycota, (**B**) *A. persici*, (**C**) *A. pomorum*, and (**D**) *L. brevis* levels. Pearson correlation coefficients (r) and associated p values are presented.

**Figure 2:**
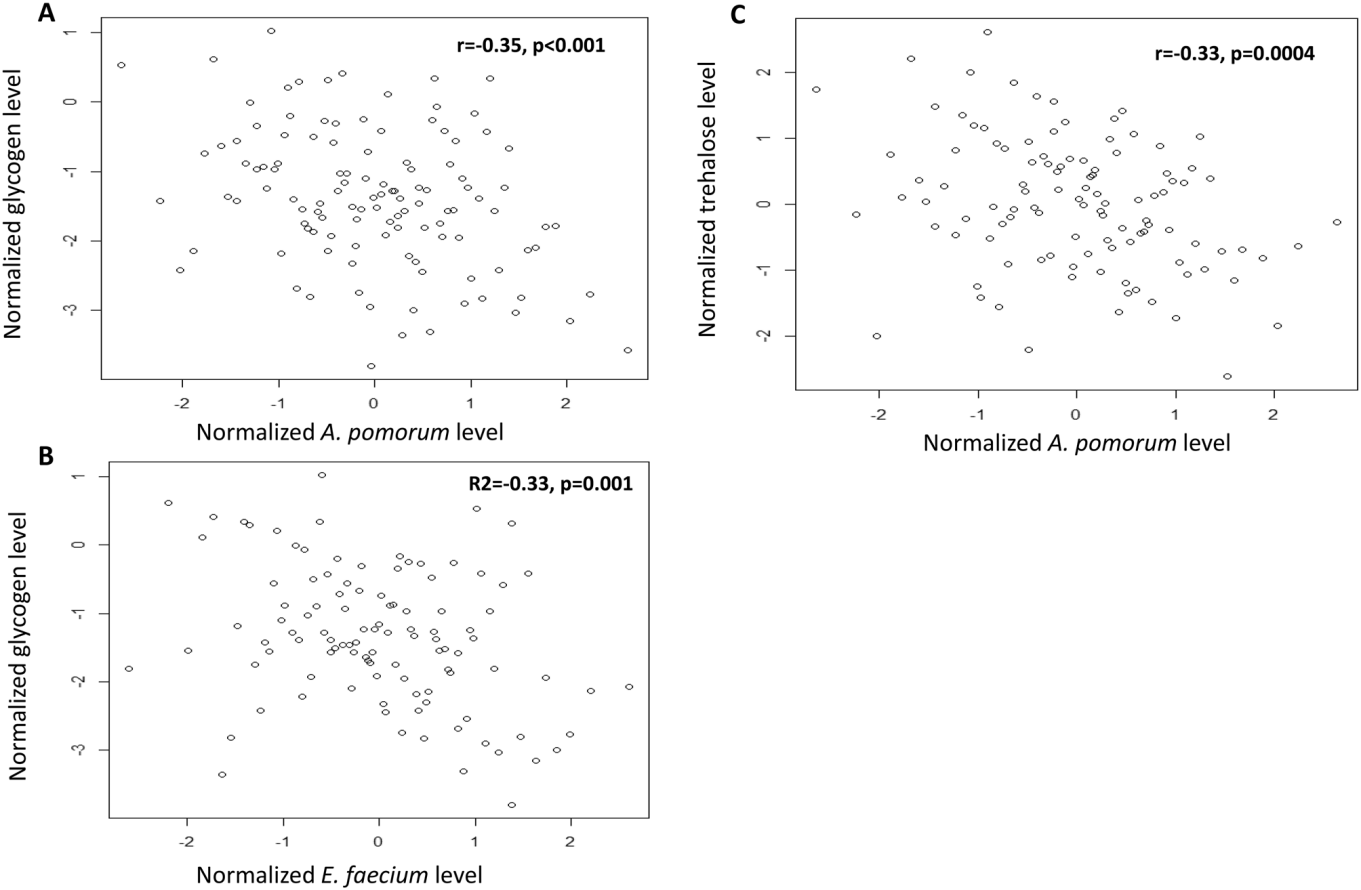
Scatterplots of normalized glycogen level versus (**A**) normalized *A. pomorum*, (**B**) *E. faecium*, (**C**) normalized trehalose level versus *A. pomorum* levels. Pearson correlation coefficients (r) and associated p values are presented.

Univariate and multivariable regression models were used to formally test the effect of fungal and bacterial taxa on metabolic pools. The negative associations between Basidiomycota, *A*. *persici*, *A*. *pomorum*, *L*. *brevis* and triglyceride levels was evident (Table 1, Fig. 1). Although the triglyceride, glycogen, and trehlose levels were observed to be highly correlated, the negative effect of these microbiome taxa on fat metabolism was independent of their effect on glycogen or trehalose metabolisms shown by the multivariable models (Table 1). Regression models also confirmed the glycogen level lowering effect of *A*. *pomorum*, and *E*. *faecium* (Table 1, Fig. 2). The negative effect of *A*. *pomorum* on trehalose levels was significant (Table 1, Fig. 2), though only in the univariate model.

**Table 1:** Univariate and multivariable regression models examining the effect of fungal and bacterial taxa on metabolite levels.

Organism	Triglyceride				Glycogen				Trehalose			
	Coef (se)	P	Coef_adj_ (se)^a^	P_adj_^a^	Coef (se)	p	Coef_adj_ (se)^a^	P_adj_^a^	Coef (se)	p	Coef_adj_ (se)^a^	P_adj_^a^
Fungi												
Total fungi	0.07 (0.11)	0.53	–	–	0.17 (0.09)	0.07	0.09 (0.09)	0.29	−0.04 (0.10)	0.68	–	–
Ascomycota	−0.02 (0.10)	0.82	–	–	0.03 (0.09)	0.75	–	–	−0.02 (0.10)	0.84	–	–
Basidiomycota	−0.23 (0.11)	0.04	−0.19 (0.09)	0.05	−0.03 (0.09)	0.74	–	–	−0.10 (0.10)	0.29	–	–
Bacteria												
Total bacteria	0.12 (0.11)	0.28	–	–	0.11 (0.09)	0.24	–	–	0.06 (0.10)	0.51	–	–
* Acetobacter persici*	−0.25 (0.10)	0.01	−0.17 (0.09)	0.07	−0.05 (0.09)	0.60	–	–	−0.11 (0.10)	0.29	–	–
* Acetobacter pomorum*	−0.68 (0.07)	< 0.0001	−0.58 (0.07)	< 0.0001	−0.35 (0.09)	< 0.0001	−0.25 (0.14)	0.07	−0.33 (0.09)	0.0004	0.11 (0.13)	0.41
* Enterococcus faecium*	−0.09 (0.12)	0.44	–	–	−0.33 (0.09)	0.0005	−0.28 (0.11)	0.01	−0.13 (0.10)	0.18	–	–
* Lactobacillus plantarum*	0.02 (0.11)	0.85	–	–	−0.02 (0.10)	0.76	–	–	0.15 (0.10)	0.14	–	–
* Lactobacillus brevis*	−0.28 (0.10)	0.009	−0.16 (0.09)	0.09	−0.14 (0.10)	0.18	–	–	−0.18 (0.11)	0.09	−0.02 (0.11)	0.81

Coef (se): regression coefficient (standard error).

^a^Multivariable regression model including triglyceride, glycogen, and trehalose metabolites in the model.

**Figure 3:**
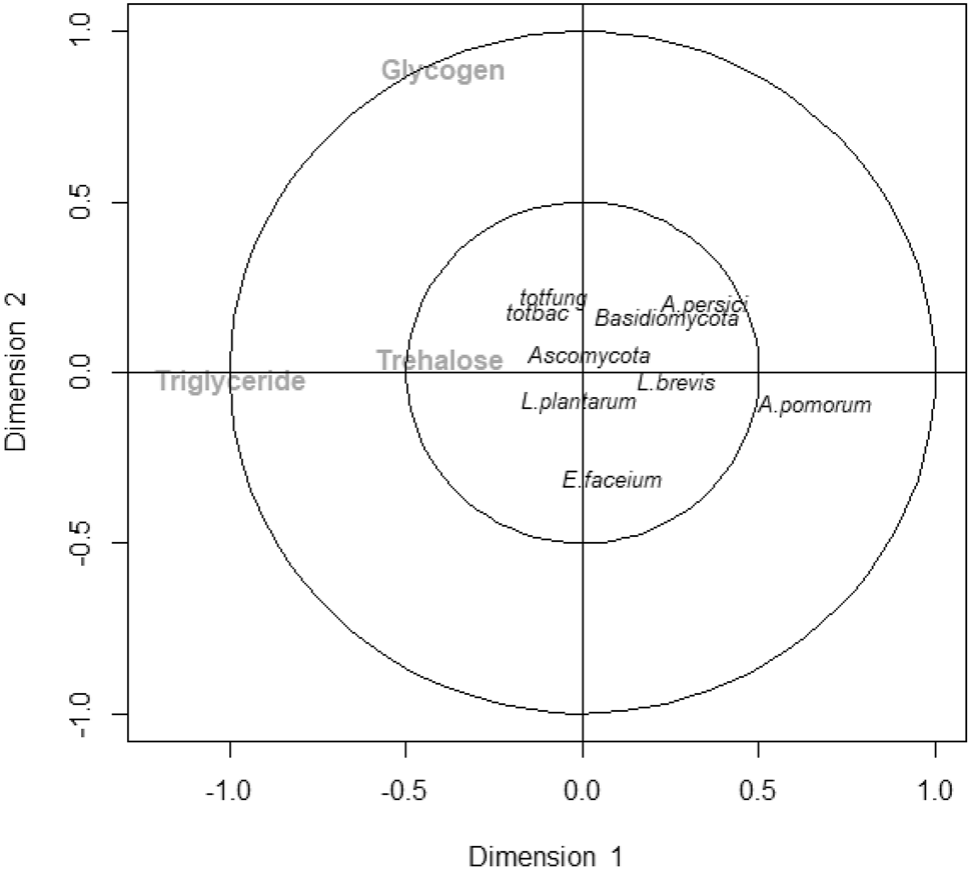
Canonical correspondence analysis for all microbiota and metabolite variables. Projection of canonical coefficients on the first two most informative canonical dimensions is presented. *TGA* triglyceride, *totalbac* total bacteria, *totfung* total fungi.

As examining the muti-way associations and interactions between microbiota and metabolite profiles (12 variables: three fungal, six bacterial, and three metabolites) via regression models is not possible with the design of this study, we adopted a mutivariate canonical correspondance analysis (CCA) to identify significant linear combinations that elucidate the most influential correlations between microbiota and metabolite profiles. Two statistically significant canonical dimensions were observed. Dimension 1 had a canonical correlation of 0.87 (p < 0.001) between metabolite and microbiota variables, while the second dimension’s canonical correlation was 0.49 (p = 0.001). Projection of canonical coefficients of metabolite and microbiota variables on the first two most informative canonical dimension showed that the first dimension primarily captured loadings separatingmicrobiota related variables and metabolites, whereas the second dimension captured loadings that further separates metabolites (such as glycogen from triglycerise and trehalose), and the different microbial taxa (Fig. 3). Controlling for all other metabolite and microbiota variables, the negative influence of Basidiomycota, *A. pomorum*, *A. persici*, and *L. brevis* on triglyceride levels was evident. Similarly, the negative effect of *E. faceium* and *A. pomorum* on glycogen levels was clear (Fig. 3). The CCA findings support the multivariable regression models that the effect of Basidiomycota, *A. pomorum*, *A. persici*, and *L. brevis* on fat metabolism is independent of glycogen and trehalose metabolism. The CCA results also support individual pairwise positive correlations between Basidiomycota, *A. pomorum*, and *A. persici*.

### Biological pathways underlying host fat metabolism, fungal and bacterial microbiota interactions

We interrogated the genomes of DGRP lines to identify host genes that influence triglyceride, Basidiomycota, *A. persici*, *A. pomorum*, and *L. brevis* levels using a genome-wide association approach. Due to many multiple tests connected, a gene-wise multiple test correction criteria of P-values less than 1 × 10^–5^ was used to filter association test results. Several genes that pass the statistical threshold criteria were identified for each phenotype (Supplemental Table 3 (sheets A–E)). We were primarily interested finding out whether there was an overlap of host genes and biological pathways that influence the triglyceride, Basidiomycota, *A. persici*, *A. pomorum*, and *L. brevis* levels. There was no overlap of genes or biological pathways that were associated with triglyceride and any of the four taxa. Three gene hits, *fipi*, *Obp57c*, and *ome* were common for Basidiomycota and *A. pomorum* levels (Supplemental Table 3 (sheets A–F)). STRING network analyses identified very limited protein–protein inreactions between rdgA, Dys, Sdc, and sfl; Ca-beta and Ih; CD43366 and CD42342 (Supplemental Fig. 7), however no biological pathways were found to be significantly enriched.

## Discussion

We aimed to investigate the relationships between fungal and bacterial microbiota, and major energy storage metabolites in *D*. *melanogaster*. We used genetically homogeneous inbred male flies of same age reared under same controlled diet and other environmental (temperature, humidity, egg and larval density) conditions. We observed that the second most common but relatively rare component of the fungal microbiota, the Basidiomycota is associated with a lower host fat metabolic pool. The most common component of the fungal microbiota, the Ascomycota did not show a significant correlation with fat, glycogen, or trehalose levels. The association of Basidiomycota with host fat metabolism was independent of glycogen and trehalose metabolisms. Through genome-wide association scans, we identified host genes and biological pathways that are associated with Basidiomycota and triglyceride levels. None of the genes and biological pathways identified to be associated with Basidiomycota and triglyceride levels overlapped, and no protein–protein and/or gene–gene interactions between the protein/gene sets were observed. These results may suggest that the association of Basidiomycota with fat metabolism could be independent of host biological pathways that control fungal microbiota or host fat metabolism. But, one cannot rule out that specific host genes may affect triglyceride levels and triglycerides can in turn affect Basidiomycota levels. Or, certain bacteria can modulate Basidiomycota levels. Clearly, further functional studies are needed, including examining the bacterial and fungal metabolites that may be modulating host metabolism.

Our findings agree with previous reports where Ascomycota taxa were reported to constitute the majority of fungal microbiota followed by Basidiomycota in insects including Drosophilids^40,54,55^. Ascomycota phylum yeasts can affect food choice^56^, reproduction^57^, and development^58,59^, and provide essential vitamins and lipids^60^ to *Drosophila*. Similar studies on the effect of Basidiomycota taxa on *Drosophila* metabolism or physiology is very limited. The extract of *Pleurotus ostreatus*, a Basidiomycota fungus, reduced circulating glucose level in the type-2 diabetes model of *D. melanogaster*^61^. A few studies with other organisms suggest a possible direct link between mycobiome and host metabolism. Members of Basidiomycota taxa correlated with fecal short-chain fatty acids and with possible effects on host metabolism in three different pig breeds^62^. In laboratory mice, gut mycobiome composition correlated with metabolic tone, where primarily members of Ascomycota showed high correlation between weight gain and triglyceride levels^63^. High-fat induced diet changed mycobiome composition and influenced obesity risk in the mice model^64,65^, however, neither the exact fungal species nor the underlying biological pathways is still not well understood.

We also observed that the bacterial microbiota members *A. pomorum*, *A. persici*, and *L. brevis* is associated with lower levels ofhost fat metabolic pool. *A. pomorum* was also associated with lower host glycogen and trehalose levels. *E. faecium* was the other bacterium that showed a negative correlation with glycogen levels. Previous reports also showed a positive correlation between *Acetobacter* and *Lactobacillus* species in *Drosophila* gut, where increased *Acetobacter* and *Lactobacillus* species decreased triglyceride levels^36,66^. *E. faecium* supplementation reduced the negative effects of high fat diet in the *Drosophila* type-2 diabetes model by decreasing the overexpression of insulin-like genes^67^. Interestingly *A. pomorum* can also modulate host metabolism through insulin signaling^32^. The variation of triglyceride content in the presence of *Acetobacter* and *Lactobacillus* is proposed to be through an increase in *Acetobacter* abundance by cocolonization with *Lactobacillus*^36^, though the exact molecular mechanism is not known. We observed significant positive correlations between Basidiomycota, and *A. persici* and *A. pomorum*, but not with *L. brevis* abundance, and all four had a significant reducing effect on triglyceride level. Three host genes *fipi*, *Obp57c*, and *ome* were identified to affect both the Basidiomycota and *A. pomorum* levels. All together, these observations suggest that host can control the abundance of certain fungal and bacterial taxa, which in turn trough interspecies and inter-kingdom interactions can modulate host fat metabolism.

There are limitations of our study. First, we do not have a species level resolution of Basidiomycota taxa to identify the actual species that affect the fat metabolism. Second, our conclusions are based on correlational observations rather than controlled interventional experimental results. Our conclusions should be tested with host fungal and bacterial microbiota manipulating controlled experiments.

In conclusion, the Basidiomycota portion of the gut fungal microbiota, *A*. *persici*, *A*. *pomorum*, and *L. brevis* are associated with host fat metabolism, thus may influence obesity risk. Future studies should also focus on fungal microbiota taxa and their interactions with the bacterial microbiota for a better and more complete understanding of how microbiome can influence obesity risk.

### Supplementary Information

Supplementary Table 3.

Supplementary Figures.

Supplementary Tables.

## Data Availability

All raw data used in the analyses are presented in Supplemental Table 3 (sheet G). Fungal and bacterial microbiota NGS sequence data is deposited to NCBI with Bioproject ID PRJNA1004984 (https://www.ncbi.nlm.nih.gov/bioproject/PRJNA1004984) (https://www.ncbi.nlm.nih.gov/Traces/study/?acc=SRP454797&o=acc_s%3Aa).
