# Supplementary material for: Basidiomycota species in Drosophila gut are associated with host fat metabolism
Source: Sci Rep. 2023 Aug 23;13:13807. doi: 10.1038/s41598-023-41027-2 (PMC10447447; doi:10.1038/s41598-023-41027-2)

Supplemental Figure Legends

Supplemental Figure 1. Relative abundance of phylum level fungal taxa in the *Drosophila melanogaster* gut. S1-S9 represent randomly selected DGRP line numbers (138, 217, 235, 26, 354, 370, 439, 705, 837, respectively).

Supplemental Figure 2. Relative abundance of phylum level bacterial taxa in the *Drosophila melanogaster* gut. S1-S10 represent randomly selected DGRP line numbers (138, 217, 235, 26, 354, 370, 439, 705, 837, 900, respectively).

Supplemental Figure 3. Histograms and horizontal box plots of (A) glucose, (B) glycogen, (C) Trehalose, and (D) Triglyceride measurements in the fly samples.

Supplemental Figure 4. Distribution (diagonal axis) of and correlations between glycogen, trehalose, triglyceride (TGA), total fungi, ascomycota, and basidiomycota levels.
*** $P < 0.001$, ** $P < 0.01$, * $P < 0.05$, · $0.1 < P < 0.05$ based on t-test statistics.

Supplemental Figure 5. Distribution (diagonal axis) of and correlations between glycogen, trehalose, triglyceride (TGA), total bacteria (Totalbac), *A. persici*, *A. pomorum*, *E. faecium*, *L. plantarum*, and *L. brevis* levels.
*** $P < 0.001$, ** $P < 0.01$, * $P < 0.05$, · $0.1 < P < 0.05$ based on t-test statistics.

Supplemental Figure 6. Distribution (diagonal axis) of and correlations between total fungi, ascomycota, basidiomycota, total bacteria (Totalbac), *A. persici*, *A. pomorum*, *E. faecium*, *L. plantarum*, and *L. brevis* levels.
*** $P < 0.001$, ** $P < 0.01$, * $P < 0.05$, · $0.1 < P < 0.05$ based on t-test statistics.

Supplemental Figure 7. Protein-protein interaction network of all genes with $P < 10^{-5}$ identified in the genome-wide association analyses. Lines between genes represent physical interactions as well as functional associations between them identified by STRING database.

Supplemental Figure 1

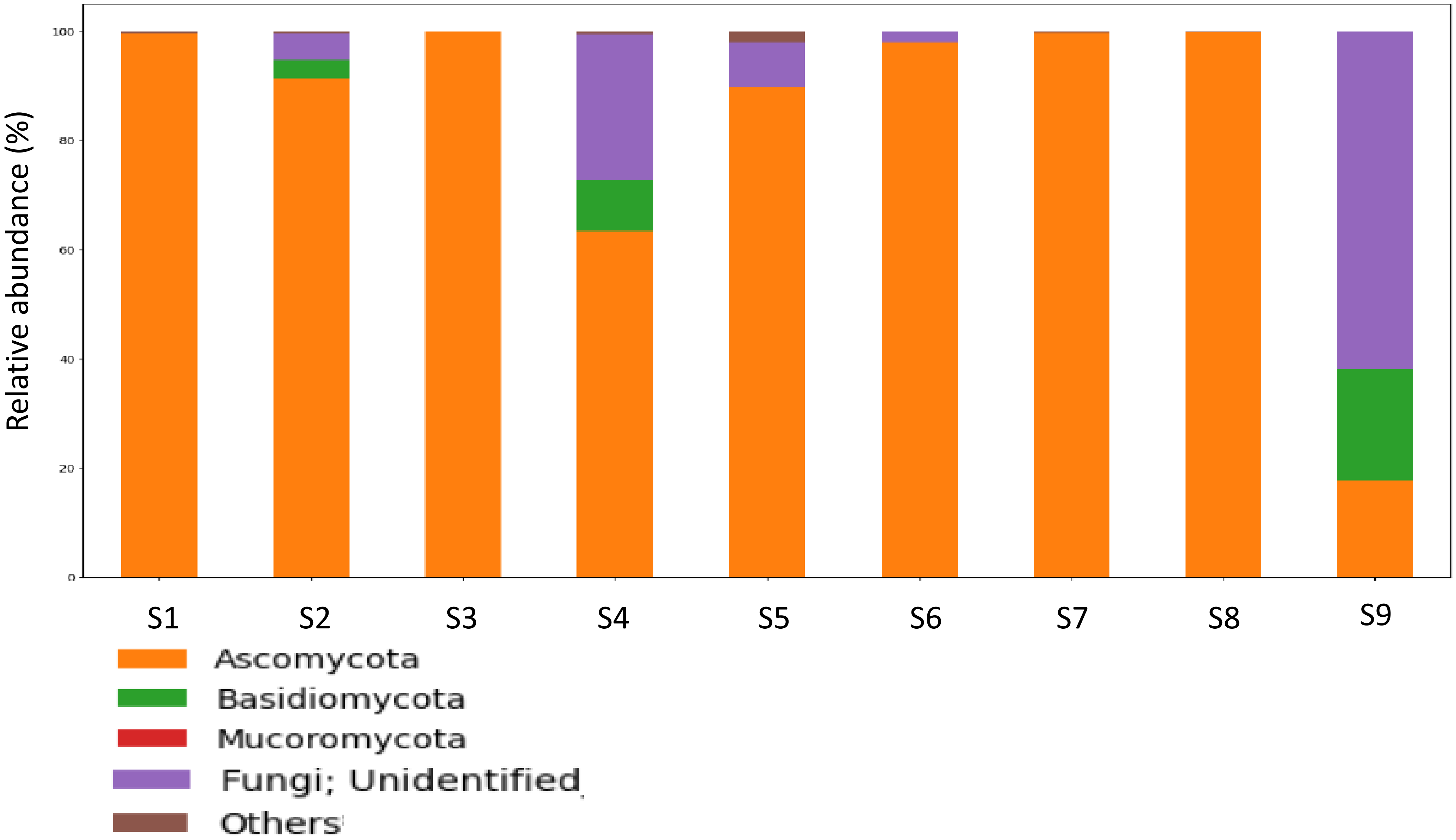

Supplemental Figure 2

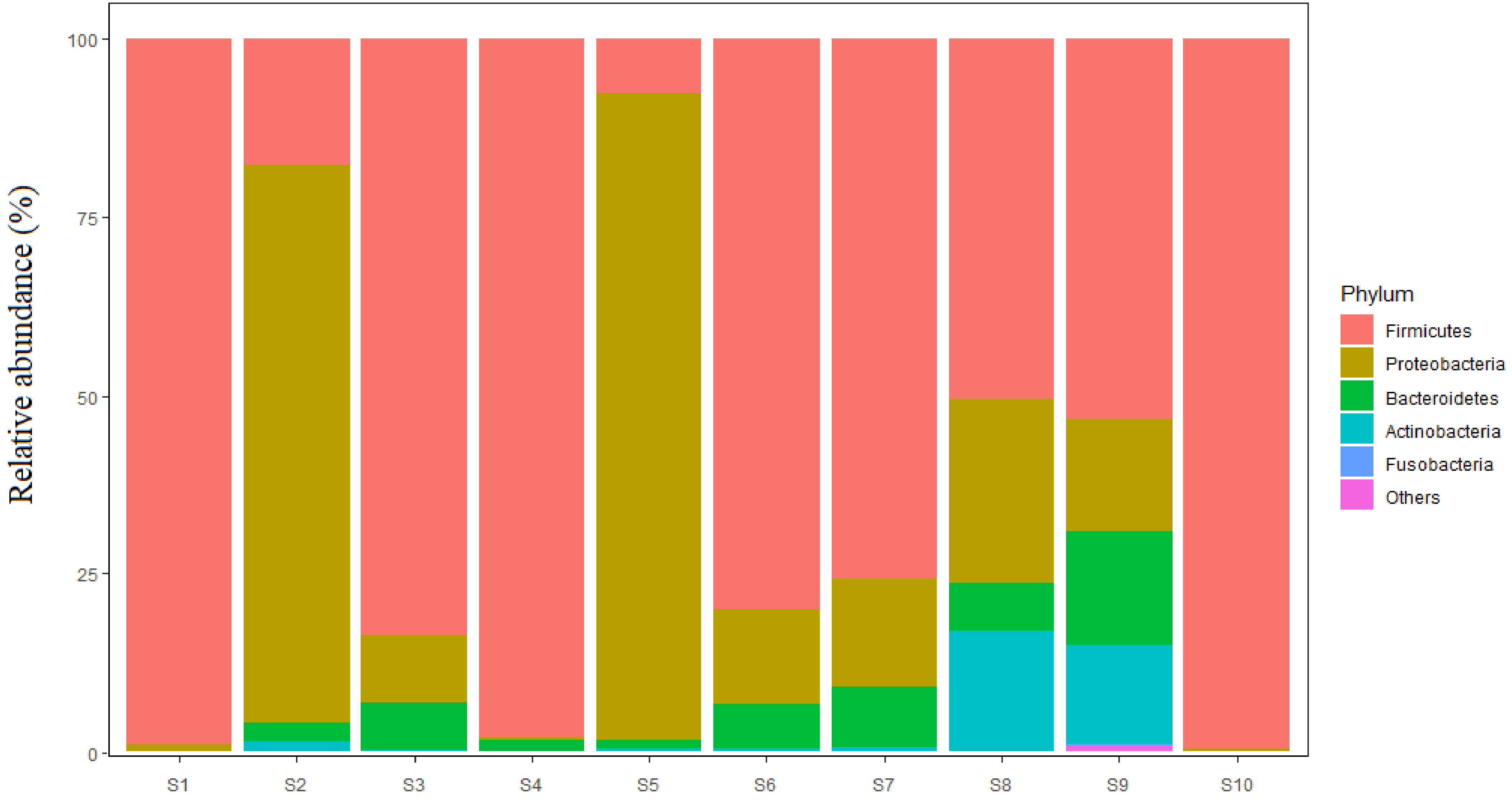

Supplemental Figure 3

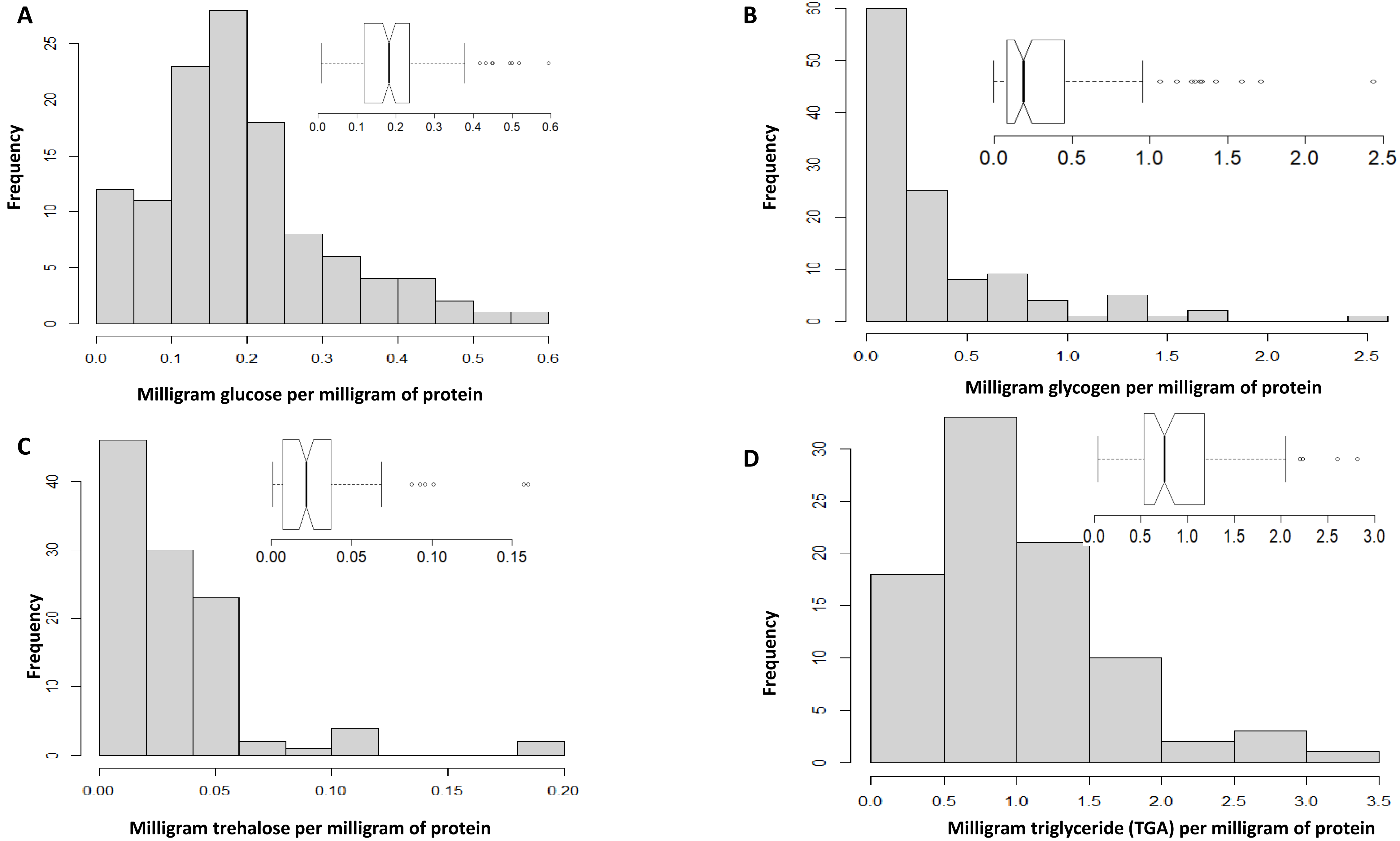

Supplemental Figure 4

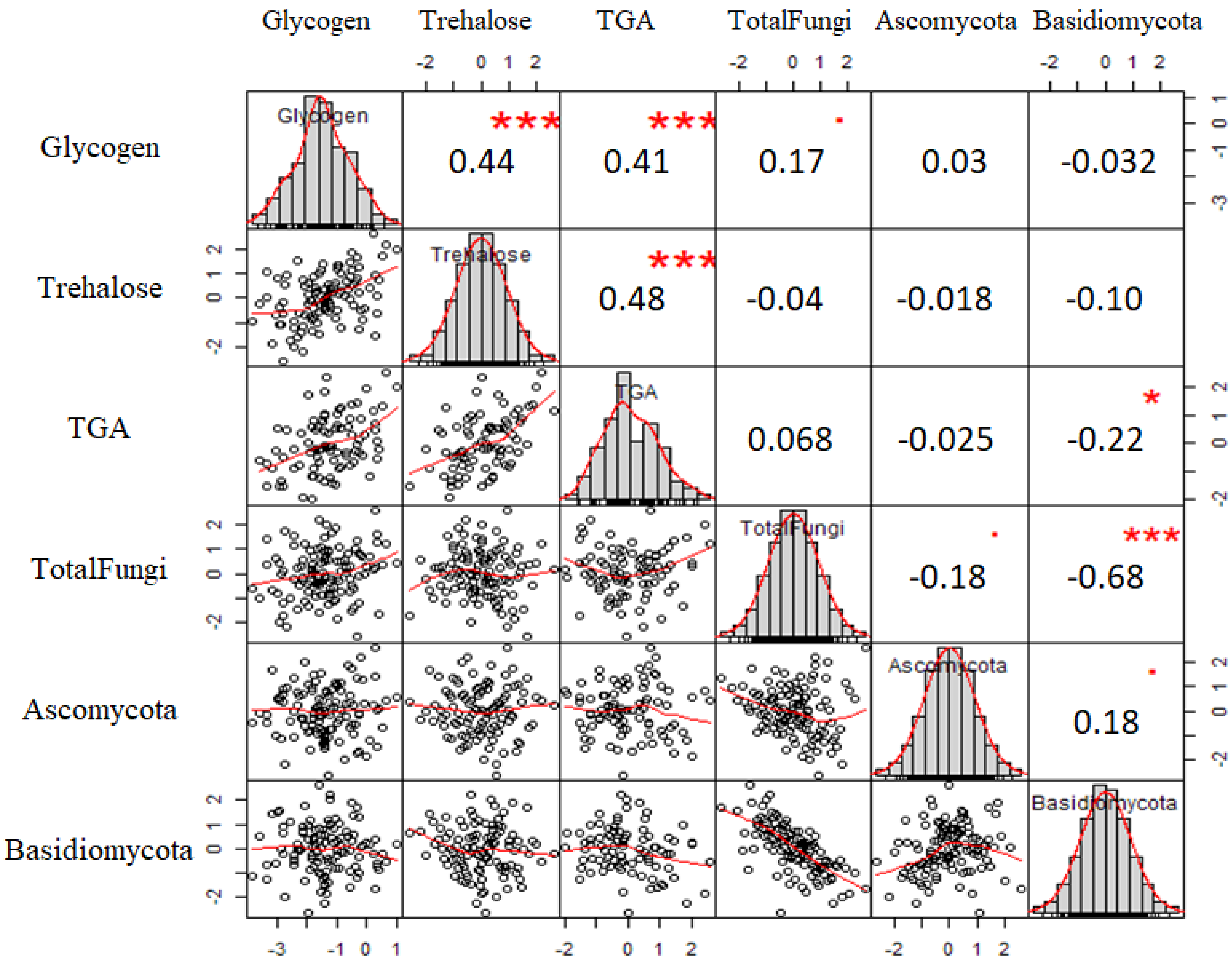

Supplemental Figure 5

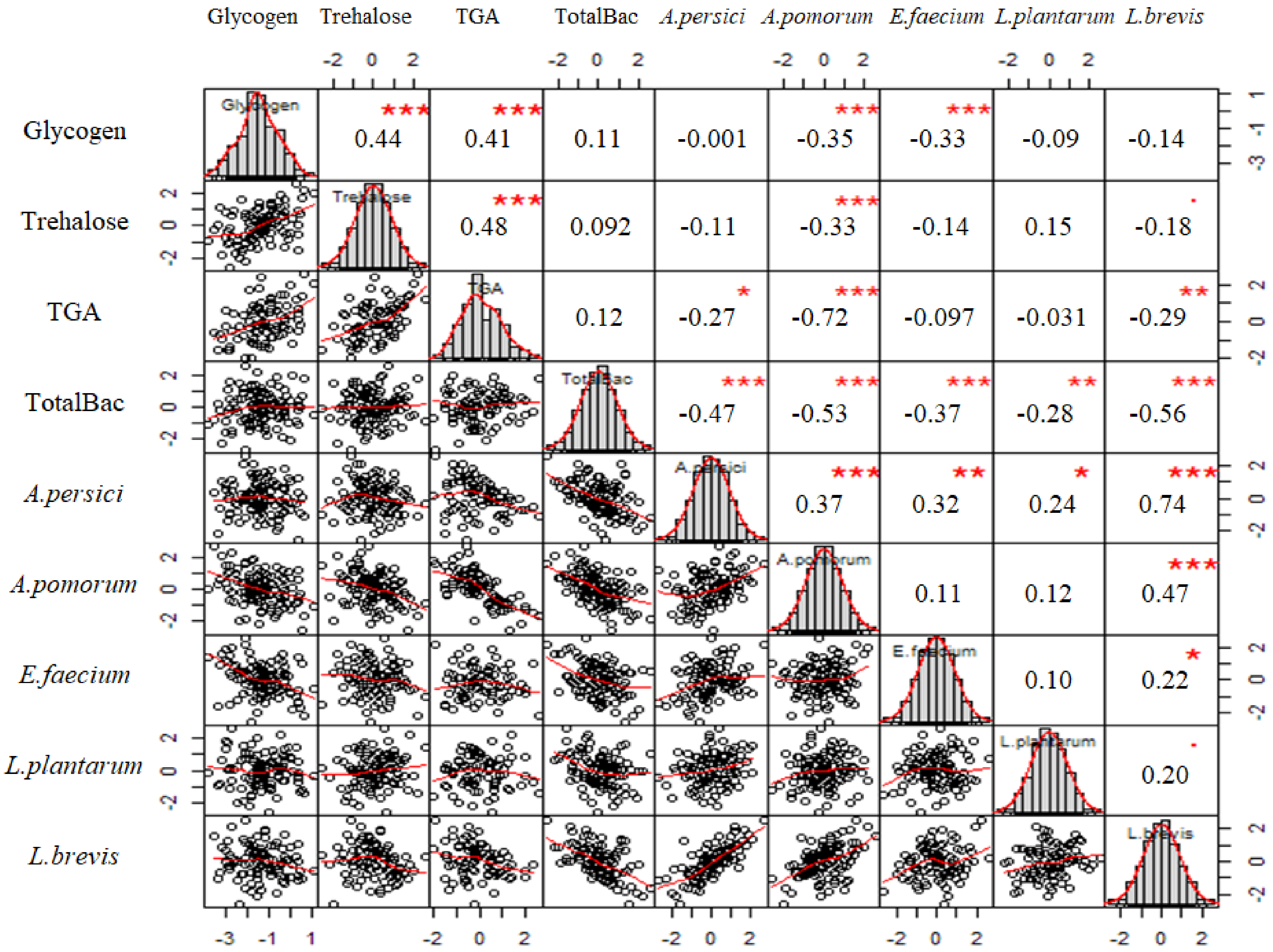

Supplemental Figure 6

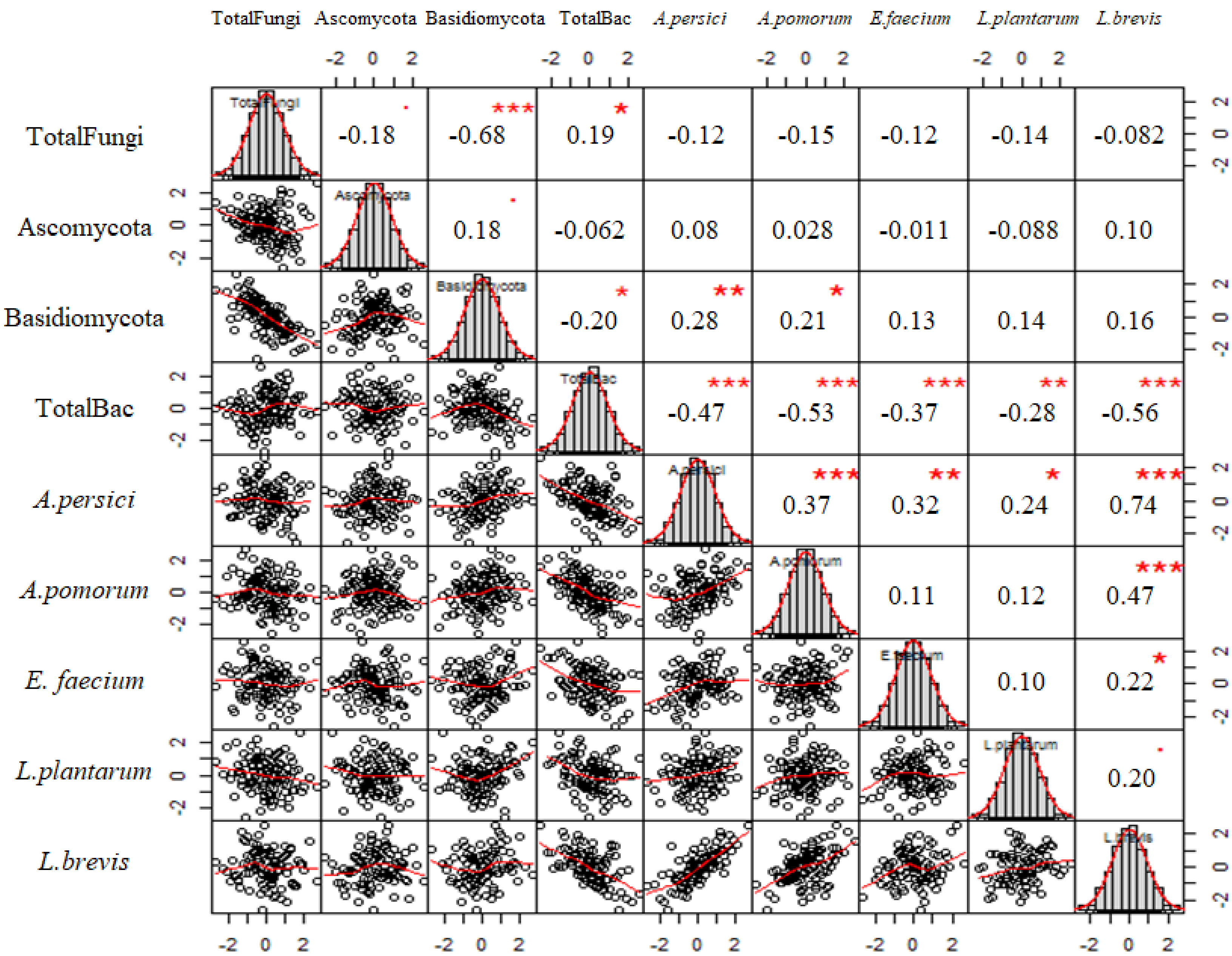

Supplemental Figure 7

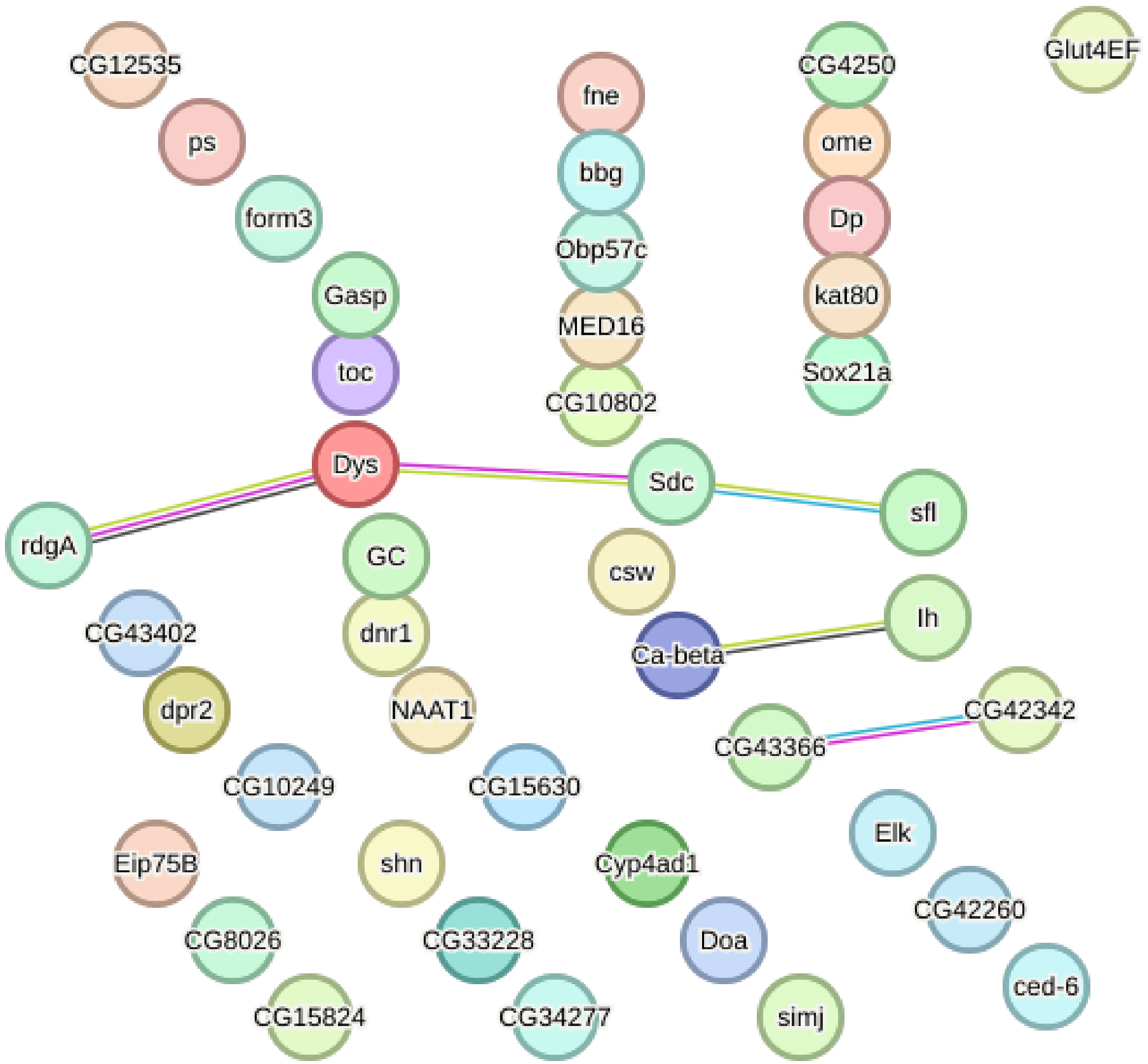

Supplement: Supplementary file 2 — Supplementary Figures. [file 41598_2023_41027_MOESM2_ESM.pdf]
