# Supplementary material for: Basidiomycota species in Drosophila gut are associated with host fat metabolism
Source: Sci Rep. 2023 Aug 23;13:13807. doi: 10.1038/s41598-023-41027-2 (PMC10447447; doi:10.1038/s41598-023-41027-2)
Supplement: Supplementary file 3 — Supplementary Tables. [file 41598_2023_41027_MOESM3_ESM.pdf]

Supplemental Table 1. Primers used in 16S rRNA and ITS region targeting and amplicon sequencing

Target Gene/Region	Amplified Region	Primers	Sequences (5'- 3')
Bacterial 16S	V3-V4	341F	CCTAYGGGRBGCASCAG
		806R	GGACTACNNGGTATCTAAT
Fungal ITS	ITS1	ITS5-1737F	GGAAGTAAAAGTCGTAACAAGG
		ITS2-2043R	GCTGCGTTCTTCATCGATGC
	ITS2	ITS3-2024F	GCATCGATGAAGAACGCAGC
		ITS4-2409R	TCCTCCGCTTATTGATATGC

Supplemental Table 2. Primers used for detection and quantification of selected bacterial and fungal taxa

Target microorganism	Genome region	DNA sequences of the primers (5′–3′)		Product size (bp)	References
Lactiplantibacillus plantarum	16s rRNA	Forward	CGAACGAACTCTGGTATTGATTG	153	(Obata et al., 2018)[1]
		Reverse	ACCATGCGGTCCAAGTTG		
Acetobacter pomorum	16s rRNA	Forward	CTAGATGTTGGGTGACTTAGTCA	204	(Wong et al., 2015)[2]
		Reverse	CGGGAAACAAACATCTCTGCTTG		
Enterococcus faecium	Genomic location	Forward	GACGGCGAAATGGGTGACT	73	This study
		Reverse	CAGAGAGTTTACGCAATGCTTGA		
Lactiplantibacillus brevis	recA	Forward	GCAGTTGCCGAGGTCCAA	64	(Xu et al., 2020)[3]
		Reverse	CCAACGCATTTTCAGCATCA		
Acetobacter persici	Genomic location	Forward	GGAGCCAGAAGCGGATTT	132	This study
		Reverse	GGTCACATACGTCATACCTGAG		
Total bacteria	16s rRNA	Forward	TCCTACGGGAGGCAGCAGT	466	(Dantoft et al., 2016)[4]
		Reverse	GGACTACCAGGGTATCTAATCCTGTT		
Ascomycota	Genomic location	Forward	GAATTGCAGMMWTCMGTGAATC	~ 200	This study
		Reverse	GCCTGTYTGAGCGTCRTTTC		
Basidiomycota	Genomic location	Forward	CGAATCTTTGAACGCAMCTTG	~ 250	This study
		Reverse	GCCTGTTTGAGTATCATGA		
Total Fungi	ITS	Forward	GGAAGTAAAAGTCGTAACAAGG	200-400	(Abliz et al, 2003)[5]
		Reverse	GCTGCGTTCTTCATCGATGC		

References

1. Obata, F., Fons, C. O. & Gould, A. P. Early-life exposure to low-dose oxidants can increase longevity via microbiome remodelling in Drosophila. *Nat. Commun.* **9**, (2018).

2. Wong, A. C. *et al.* The Host as the Driver of the Microbiota in the Gut and External Environment of Drosophila melanogaster. *Appl. Environ. Microbiol.* **81**, 6232–6240 (2015).

3. Xu, Y. *et al.* EGCG ameliorates neuronal and behavioral defects by remodeling gut microbiota and TotM expression in Drosophila models of Parkinson's disease. *FASEB J.* **34**, 5931–5950 (2020).

4. Dantoft, W., Lundin, D., Esfahani, S. S. & Engström, Y. The POU/Oct Transcription Factor Pdm1/nub Is Necessary for a Beneficial Gut Microbiota and Normal Lifespan of Drosophila. *J. Innate Immun.* **8**, 412–426 (2016).

5. Abliz, P., Fukushima, K., Takizawa, K., Miyaji, M. & Nishimura, K. Specific oligonucleotide primers for identification of Hortaea werneckii, a causative agent of tinea nigra. *Diagn. Microbiol. Infect. Dis.* **46**, 89–93 (2003).
